# Academy of Stomatology

**Published:** 1904-11

**Authors:** 

**Affiliations:** Academy of Stomatology


### Reports of Society Meetings.

### ACADEMY OF STOMATOLOGY.

A REGULAR monthly meeting of the Philadelphia Academy of Stomatology was held at its rooms, 1731 Chestnut Street, on the evening of Tuesday, June 28, 1904, the President, Dr. I. N. Broomell, in the chair. The evening was occupied with the consideration of incidents of practice.

*Dr. M. Schamberg.*—There is a case which should be reported by Dr. Fogg. He brought to my office about a week ago a patient who had a rather peculiar condition upon the root of a central incisor tooth. He noticed a swelling upon the gum overlying the root of that tooth, and upon testing it found it to be vital and decided that the condition must be a pericemental abscess. He was very anxious to find some cause for the trouble, and a radiograph was taken, which showed a large area of tooth destruction. This destruction indicated resorption. The tooth was kept under observation for quite a while. Yesterday the doctor told me that, in addition to this tooth, quite a number of the other teeth exhibited this irregular resorption, to such an extent, indeed, that the tooth-crown was lost. It is the only case of the kind I have ever seen, and it is of such an unusual type that I thought it might be interesting for the Academy to hear of the case. I would be very glad to know whether any have met with a similar condition.

*Dr. E. T. Darby.*—Were they all vital teeth?

*Dr. Schamberg.*—They were in the main vital. In some of the lower teeth the pulps were dead.

*Dr. James Truman.*—How old was the patient?

*Dr. Schamberg.*—The patient was about thirty-two or thirty-five years old.

*Dr. H. R. D. Swing.*—Was there any pyorrhœa?

*Dr. Schamberg.*—There was no pyorrhœa, but there was a certain amount of recession. The tooth was vital up to the time of its removal.

*Dr. I. N. Broomell.*—Had the alveolar bone filled in the space?

*Dr. Schamberg.*—No; the alveolar process in most cases was resorbed over the site of the root resorption, due more to infection which reached the part in consequence of the food débris entering the spaces. Almost every surface of the molar teeth affected showed resorption.

REPORT OF A CASE.

*Dr. A. N. Gaylord.*—I had an interesting case some time ago of a gentleman upward of sixty, who called upon me and said that two and a half years previously he had a badly inflamed jaw and face and was unable to open his mouth. He was fed through a tube. He went to a physician, who stated that he had a malignant growth and suggested that he go to a specialist. The specialist concurred in the opinion and sent him to a surgeon for operation. The tissues were laid open, the bone scraped, and the man told that he would recover. From that time the man had a sinus discharging on the border of the jaw. Fortunately he had a heavy growth of whiskers, which covered it, but he was obliged to carry absorbent cotton to keep it in a respectable condition. The history was the familiar one of alveolar abscess, although there was nothing on the inside of the mouth to indicate one. There was no sign of an embedded root, although I suspected one, and suggested that the man have a skiagraph taken. The first one taken of the entire jaw showed a faint outline of a root. That was not satisfactory, and I had a second one taken, using a film on the side of the border of the jaw, and that showed a root plainly. I made an application of cocaine and cut down upon it, but it was too firmly embedded to be removed by the forceps. I used a surgical burr, took the root out, and passed a probe from the opening on the outside of the face through the tissues and found that it came out through an opening in the bone. This was washed out with antiseptics. There was no discharge the next day, and on the second day the opening on the face had closed up. I opened it again, fearing that it had closed too quickly, but from the third day the wound has remained closed and there has been no discharge. I saw the patient this afternoon. The inside of the mouth presents an entirely healthy appearance and the wound is entirely healed. For this credit is due to the X-ray diagnosis.

*Dr. E. T. Darby.*—Dr. Gaylord's narrative of that case reminds me of one I had a few years ago, which I mention to show that we may be sometimes mistaken about the vitality or non-vitality of a tooth. Some years ago a gentleman was brought to me by his dentist, and the facts of the case were these: The man was a fine healthy specimen of about thirty-five or more. He had had a discharge upon the jaw opposite the bicuspid or molar tooth for a year or more. He had consulted his family physician, who told him that his blood was diseased, and had given him remedies. He was obliged to wear a black patch for the discharging sinus. He was travelling one day in one of the New England States, and a gentleman sitting behind him leaned forward and said, " My friend, that comes from a tooth." He said, " You are mistaken; my physician in Philadelphia tells me it is from the condition of my blood." The reply was, " Well, I am a surgeon. I simply say that comes from a tooth." The gentleman was a little indignant, but when he came to Philadelphia he consulted Dr. Agnew, who said, " I think he told you the truth," and referred him to me. The gentleman brought his dentist with him for a consultation. The dentist began by saying, " I know, doctor, this does not come from his teeth; I have examined them over and over and over again." I looked into the patient's mouth and opened the right lower second bicuspid. I passed in a probe, and pus welled up. The dentist had been for thirty years in practice and had never seen one of these cases. I advised extraction, which was done, and three days later I met the gentleman, who said there had not been a particle of pus since.

I mention this to show that a man with thirty years of experience had never happened to see an abscess discharging at the angle of the jaw from a lower molar or bicuspid tooth, and when he had it before him he did not recognize it. One can hardly conceive of that happening. The surgeon in that instance was brighter than the dentist. In Dr. Gaylord's case the dentist was brighter than the surgeon.

*Dr. P. B. McCullough.*—Four or five years ago I mentioned a case which is in accord with the present discussion. It is already recorded in the transactions of the Academy. I was asked by a veterinary surgeon to examine the mouth of a dog with an abscess discharging below the line of the lower jaw. Such cases were so common in dentistry that I suspected an abscess. I examined all the teeth, but could find no decayed tooth. I felt that the only way to diagnose the condition was to allow the probe to be a guide as it passed through the fistulous tract. The abscess was in the neighborhood of the first or second lower molar. I called the attention of the veterinary surgeon to this, and he recommended extraction. I assumed that he should know more about the anatomy of the tooth of the dog. The tooth was extracted and found to be devitalized.

I would like to mention some experiments made with plaster. There is soon to be published in the INTERNATIONAL DENTAL JOURNAL a *résumé* of these experiments in practical work. There has been much written about the difficulty of working with plaster because of its different stages of expansion. The Academy will remember that work of this kind dates far back into dentistry. Recently Dr. Spencer announced that he had a plaster which was non-expanding. I have made no experiments with his product, and therefore do not know of its value. I have, however, discovered a plaster which is absolutely non-expanding. I mention in this article to be published how my attention was first called to it and the circumstances that led up to the discovery of the trick that makes the non-expanding plaster. The method consists of the proper treatment of lime-water. The water must be boiled. Unslaked lime is added and the clear liquid is drained off. If a fraction of salt is added the plaster will expand. If boiled water is used alone the plaster will expand. There was no difference in the degree of expansion from all the combinations I tried. I have soaked the plaster in water several days after it was mixed and then vulcanized it into the tube, but without any change. I also made another test with gum-arabic water, without boiling, and in this case there was no expansion. This is not of much value for the reason that the gum-arabic is so readily soluble in water that I felt that in vulcanizing it might soften.

*Dr. James Truman.*—I think the report of Dr. McCullough is very important. I would like him to explain his use of the glass tube with the plaster made with lime-water boiled.

*Dr. McCullough.*—I will say, briefly, that the first test that I made was with lime-water that had been boiled for domestic use. In making some further tests I noticed that the first test was made with boiled water, the second, without boiling. I then went through a series of experiments. All of the tubes cracked within twelve minutes, except the one with the plaster which had been mixed with the boiled lime-water. This is interesting from the stand-point of the chemist. I do not know that the chemistry of plaster is known. I remember being taught that it was a problem that was not understood. I am inclined to think that it can be understood, but why boiled lime-water should affect plaster as it does I do not know. It occurred to me as possible that the water being deoxygenated by boiling would account for it, but the fact that I tested boiled water without the lime shows the error of this. The water was sterile without the addition of lime. With gum-arabic water the plaster did not expand, yet that water was not boiled.

*Dr. Schamberg.*—Dr. McCullough's report is very interesting, and the discovery should be of much value. One thing occurred to me when he spoke of water losing its oxygen through boiling. When I was in Porto Rico we boiled the water for drinking, and we found it very flat. With an ordinary bicycle tube which we placed in the water we forced the air through it. In that way it took up a certain amount of oxygen. It occurred to me that Dr. McCullough might try that method with his experiments to find out whether boiled water so treated would influence the expansion of the plaster.

Dr. Gaylord's remarks interested me particularly, because I have recently had three cases where a fistulous tract was established upon the line or beneath the lower jaw, and due to abscessed teeth. In many instances that have come to my attention prior to this time I have noticed that it was largely due to the advice of the medical practitioners, and occasionally the dentist is at fault. This is not surprising when we consider how hard it is to test a tooth for vitality. When an obscure case comes into my office I always use the rubber dam and test each tooth separately by the use of the ethyl chloride spray. I have been at times much humiliated, after advising dentists that certain teeth were not vital, to find that they were vital. I have concluded that often there is a loss of sensation in tooth-pulps without loss of vitality. The sensory nerves may have lost their function, but there is a certain amount of circulation in the pulp, and the pulp is vital. We can conceive of such a thing being possible when we realize that we may have a palsied arm and yet have circulation in that arm. So in several cases where I thought I was at the bottom of some troublesome neuralgia and the tooth has been opened, the dentist has found a vital pulp. I have, therefore, hesitated much more about advising the drilling into teeth when they give no sensation upon the application of the cold test. I believe it is a good thing to try the transillumination method. When there has been doubt as to whether there was a small apical inflammation, I have followed up these tests with the X-ray, and in many instances have noticed the most incipient abscess where we might scarcely expect to find a drop of pus. We are all familiar with the immense amount of cellulitis involving the face when there has been less than a drop of pus at the apical space.

*Dr. McCullough.*—Dr. Schamberg's reference to the pulp having physiological function without sensation recalls to me a case of a woman for whom I had to drill into the labial face of a cuspid tooth for arsenical application. I knew that if she had the slightest pain she would tell me. I drilled into that tooth until I reached the pulp, and yet there was no blood and she felt no pain whatsoever. The tooth was without cavity or decay. As soon as I struck the pulp-cavity I got blood.

In another case I moved the entire upper set of teeth in a girl when she was quite young. The extent to which the central incisors had to be moved to articulate was so great that it took two years to move them. They were very slowly moved a certain distance during the winter months, and then retained with a fixed appliance. In the summer there was no change made. In the winter the teeth were again moved. Several years afterwards the girl complained that her teeth were turning yellow. I asked several men if they thought they were devitalized. The interesting point is that these teeth for a period of four years after completion of the operation showed no change.

*Dr. James Truman.*—I can appreciate very fully the difficulty of diagnosing a devitalized pulp. Ethyl chloride is one of the best things for the purpose, but I think it is often fallacious; so is ice. I am well aware of Dr. Jack's rather exhaustive formula, but I have not been as successful as he has in that direction. I have had as much satisfaction with the galvanic current as with any other method. I often wonder that it is not more generally used. It seems to me more exact than the cold test. My experience, however, is not sufficiently large to prove that as a fact; in my hands it has worked well.

Otto E. Inglis,
*Editor Academy of Stomatology.*

